# Crystal structure of SARS-CoV-2 nsp10/nsp16 2′-O-methylase and its implication on antiviral drug design

**DOI:** 10.1038/s41392-020-00241-4

**Published:** 2020-07-29

**Authors:** Sheng Lin, Hua Chen, Fei Ye, Zimin Chen, Fanli Yang, Yue Zheng, Yu Cao, Jingxin Qiao, Shengyong Yang, Guangwen Lu

**Affiliations:** 1 West China Hospital Emergency Department (WCHED), State Key Laboratory of Biotherapy and Cancer Center, West China Hospital and Collaborative Innovation Center of Biotherapy, Sichuan University, 610041 Chengdu, Sichuan China; 2 Disaster Medicine Center, West China Hospital, Sichuan University, 610041 Chengdu, Sichuan China

**Keywords:** Microbiology, Structural biology

**Dear Editor**,

The unexpected outbreak of a novel human coronavirus infection has imposed great threat to public health. Thus far, this newly-identified virus has spread to 215 countries and territories, infected more than 3.5 million people, and caused over 240,000 deaths worldwide.^1^ Although intensified countermeasures have been implemented globally to control the virus infection, the pandemic is still surging with a daily increase in infection case of over 65,000 ever since April 1st 2020.^1^ No prophylactic vaccines or clinical drugs are currently available to prevent or treat the disease, namely coronavirus disease 2019 (COVID-19).

Coronavirus is a group of enveloped positive-sensed RNA viruses that replicate in host cell cytoplasm via a large membrane-associated RNA replication/transcription machinery comprising at least sixteen virus-encoded non-structural proteins (nsp1 to nsp16). Among these, nsp10 and nsp16 form a protein complex, which functions to catalyze the methylation of the penultimate nucleotide of the viral RNA cap at the ribose 2′-O position. Such methylation process would convert the RNA cap of the virus from a cap-0 structure (featured as ^7Me^GpppA…) into a cap-1 structure (featured as ^7Me^GpppA_2′__-O-Me_…) to mimic cellular mRNAs and thereby to prevent recognition of viral RNAs by host innate immunity.^2,3^ In order to learn the structural features of SARS-CoV-2 nsp10/nsp16 2′-O-methylase, the two proteins were co-expressed in *Escherichia coli* (*E. coli*) and purified as a stable heterodimer complex in solution (Supplementary Fig. S1a). The protein complex was then crystallized in the presence of S-adenosyl-l-methionine (SAM, a methyl donor for the methylation reaction) supplemented at a 1:5 protein-to-SAM molar ratio. The structure was solved at 2.5 Å resolution. We also crystallized nsp10/nsp16 without additional SAM and solved its structure at 2.8 Å resolution (Supplementary Table S1). In each case, the solved structure contains a single 1:1 bound nsp10/nsp16 hetero-complex in the crystallographic asymmetric unit. In addition, the electron densities for the SAM molecule were clearly observed in both structures (Supplementary Fig. S2a, b), demonstrating that the protein had captured SAM simultaneously when expressed in *E. coli*. As expected, the two structures solved in this study are quite similar to each other, showing a root mean square deviation (RMSD) of ~0.2 Å for 115 equivalent nsp10 Cαs and of ~0.2 Å for 298 nsp16 Cαs, respectively (Supplementary Fig. S2c).

Overall, the nsp10/nsp16 structure can be viewed as an nsp16 monomer perching on top of an nsp10 molecule (Fig. 1a). The top-residing nsp16 is composed of twelve β-strands, seven α-helices, and five 3_10_ helices (Supplementary Fig. S3a). These secondary structural elements assemble into a compact fold. The core of the structure is made of strands β1–β7 and helices αZ, αA, αD, and αE. This central core is further decorated at the protein N-terminus by the η1 3_10_-helix, β8-strand, and αf-helix, between strands β3 and β4 by the β9-strand and η2 3_10_-helix, between strand β4 and helix αD by the η3 3_10_-helix, and at the C-terminus by strands β10–β12, α-helices αg and αh, and 3_10_-helices η4 and η5. The bottom-sitting nsp10 comprises three β-strands, three α-helices and two 3_10_ helices (Supplementary Fig. S3b). Strands β1′–β3′ form a central β-sheet, and the helical components (α-helices α2′–α4′ and 3_10_-helices η1′ and η2′) are packed together to cover one surface of the central sheet (Fig. 1a). Both the individual protein-subunit structure and the protein-subunit interaction mode of the SARS-CoV-2 nsp10/nsp16 complex resemble those observed in other beta-coronaviruses. Superimposition of our structure onto those of SARS-CoV (PDB: 3R24) and MERS-CoV (PDB: 5YN5) revealed an RMSD of ~0.9-Å (for 291 Cα-pairs) and ~0.8-Å (289 Cα-pairs) for nsp16 and of ~0.5-Å (109 Cα-pairs) and ~0.8-Å (110 Cα-pairs) for nsp10, respectively (Fig. 1b). Obvious conformational difference, however, was observed for the αf/αZ interloop as well as the η3-helix and its following loop (designated as η3-loop) in nsp16. In addition, the α1′-helix of nsp10 was completely density-untraceable in our structure, which can be clearly observed in both SARS-CoV and MERS-CoV nsp10 (Fig. 1b).

**Fig. 1:**
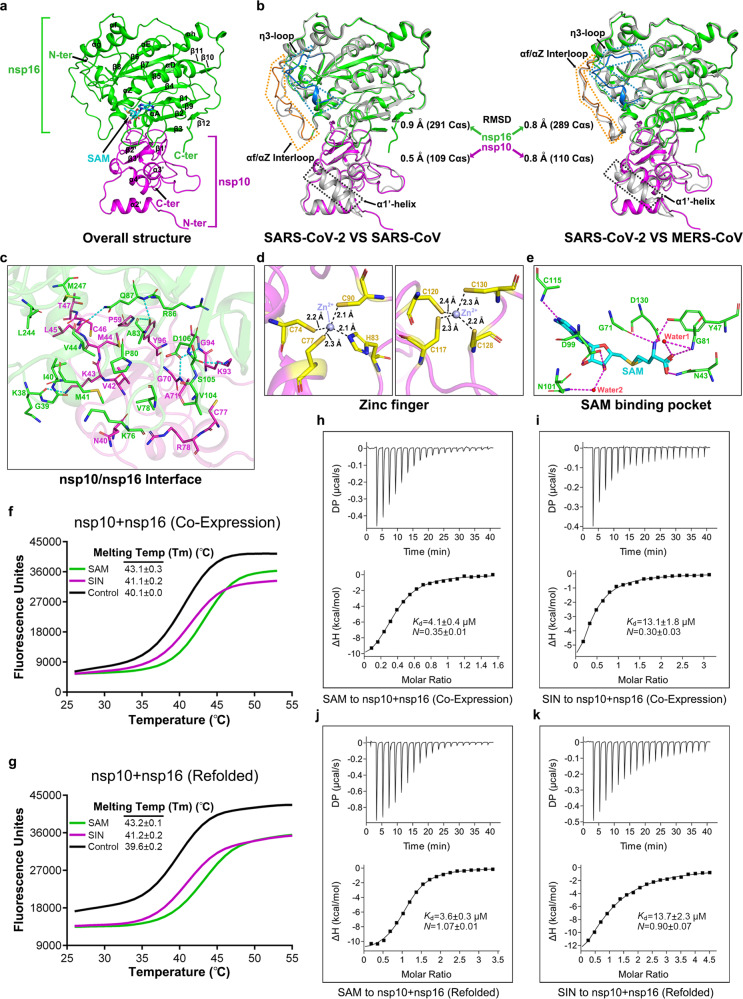
Structure of SARS-CoV-2 nsp10/nsp16 hetero-dimer. **a** Overall structure of the hetero-complex formed between nsp10 (magenta) and nsp16 (green). The secondary structural elements and the bound SAM molecule are labeled. **b** Superimposition of the nsp10/nsp16 structure of SARS-CoV-2 onto those of SARS-CoV (left panel, PDB: 3R24) and MERS-CoV (right panel, PDB: 5YN5). The color scheme for our structure is the same as in **a**, and the SARS-CoV and MERS-CoV structures are shown in gray. Those elements exhibiting variant conformations are highlighted and marked. **c** Detailed interactions at the nsp10/nsp16 binding interface. Dashed lines indicate hydrogen-bonds. **d** A magnified view on the two zinc-finger motifs in nsp10. **e** Detailed interactions between SAM and nsp16 at the SAM-binding pocket. **f**, **g** Interaction of SAM and SIN with SARS-CoV-2 nsp10/nsp16 characterized by differential scanning fluorimetry (DSF). The DSF data obtained with the protein-complex purified directly from *E. coli* (designated as co-expression) and the protein-complex after the denaturing-refolding cycle (designated as refolded) are shown in **f** and **g**, respectively. **h**–**k** Binding of SAM and SIN to SARS-CoV-2 nsp10/nsp16 characterized by isothermal titration calorimetry (ITC). **h** SAM to nsp10/nsp16 (co-expression). **i** SIN to nsp10/nsp16 (co-expression). **j** SAM to nsp10/nsp16 (refolded). **k** SIN to nsp10/nsp16 (refolded)

Facilitated by the solved structure, we then characterized the key structural elements involved in the nsp10/nsp16 function. 1. Within the heterodimer complex, nsp10 is indispensable by acting as a co-factor for the nsp16 methylase.^2,3^ This intimate interaction is mediated by a total of 31 (N40, V42, K43, M44, L45, C46, T47, P59, G70, A71, C77, R78, K93, G94, and Y96 in nsp10 and K38, G39, I40, M41, V44, K76, V78, P80, A83, R86, Q87, V104, S105, D106, L244, and M247 in nsp16) residues, forming an extended interface with a buried surface area of ~933 Å^2^ and ~872 Å^2^ in nsp10 and nsp16, respectively (Fig. 1c). 2. Within nsp10, two zinc-fingers are observed to form to stabilize the nsp10 structure. One zinc ion is coordinated by residues C74, C77, H83, and C90, and the other by C117, C120, C128, and C130 (Fig. 1d). 3. Within nsp16, an SAM-binding pocket is constituted by amino acids N43, Y47, G71, G81, D99, N101, C115, and D130. These residues and the neighboring water molecules form a hydrogen-bond network to trap the bound SAM in an extended conformation (Fig. 1e). All these SAM-engaging residues are conserved in SARS-CoV and MERS-CoV, highlighting a conserved SAM-binding mode in these viruses (Supplementary Fig. S3a). The conserved SAM-interaction mode also highlights the possibility of developing a pan-antiviral inhibitors by targeting this SAM-binding pocket. 4. Within nsp16, a putative RNA-substrate binding groove is lined on one side by the αf/αZ interloop and on the other by the η3-loop. These components have created an extended solvent-exposed groove with a strong positive electrostatic surface (Supplementary Fig. S4a). Notably, an intergroove bridge was observed to form between Y30 and K137 in our structure, which parallels our structure more to a recently-solved SARS-CoV-2 nsp10/nsp16 structure with a bound ^7Me^GpppA cap (PDB: 6WKS) and the structure of MERS-CoV nsp10/nsp16 in complex with an RNA-cap substrate (PDB: 5YNM) than to the SARS-CoV nsp10/nsp16 structure (PDB: 3R24) whose RNA-binding groove is completely open (Supplementary Fig. S4a). This may indicate that our structure has accidentally trapped the groove in a post RNA binding conformation during crystallization, though the RNA-substrate is absent. The observations also highlight the plasticity of the RNA-binding groove in nsp16. Accordingly, previous studies on SARS-CoV nsp10/nsp16 indeed showed that the groove-lining residues are of high flexibility, showing either weak electron-densities or high B factors.^2,3^ It is also notable that a recent study reporting the SARS-CoV-2 nsp10/nsp16 structure in the ternary state with bound SAM and ^7Me^GpppA has proposed a large conformational change associated with cap-binding.^4^ The fact that we have been able to trap the cap-binding groove in a post-RNA binding conformation without the bound RNA-cap substrate further suggests that the proposed conformational change is unlikely a substrate-binding induced event but rather the result of the intrinsic plasticity of the RNA-binding groove.

We finally investigated the interactions of SARS-CoV-2 nsp10/nsp16 with SAM, sinefugin (SIN), and the ^7Me^GpppA RNA-cap substrate in solution. Noted that nsp10/nsp16 likely has simultaneously captured SAM in its SAM-binding pocket during co-expression in *E. coli*, we further denatured the protein heterodimer to remove any residual SAM and then refolded the protein complex to obtain nsp10/nsp16 without pre-bound SAM (Supplementary Fig. S1b). The protein preparations (co-expression and refolded) were then used in parallel in the differential scanning fluorimetry (DSF) and isothermal titration calorimetry (ITC) assays. Potent interactions were expectedly observed for both SAM and SIN. The calculated shift in melting-temperature for SIN, however, was lower than that for SAM by ~2° (Fig. 1f, g). The ITC data further showed that SIN binds to SARS-CoV-2 nsp10/nsp16 (13.1 ± 1.8 μM and 13.7 ± 2.3 μM to the co-expression and refolded proteins, respectively) with an over three-fold lower affinity than SAM (4.1 ± 0.4 μM and 3.6 ± 0.3 μM to the co-expression and refolded proteins, respectively) (Fig. 1h–k). It is also notable that the stoichiometry calculated with the protein purified directly from *E. coli* were ~0.35 for SAM and ~0.3 for SIN, indicating that about 2/3 of the SAM-binding pockets were pre-occupied during expression. These values were determined to be ~1.07 and ~0.9 with the refolded protein, well echoing our structural observation showing that the heterodimer contains a single SAM-binding pocket in nsp16 (Fig. 1h–k). The binding affinity between SARS-CoV-2 nsp10/nsp16 and the ^7Me^GpppA RNA-cap substrate was also investigated via ITC, which was determined to be 9.4 ± 2.1 μM (Supplementary Fig. S4b). Mutation of the bridging residues Y30 and K137 in nsp16 into Ala could abolish such binding. While the double mutation did not affect the solution gel-filtration behavior of the nsp10/nsp16 complex (Supplementary Fig. S1c), no direct interactions were detected in ITC for the Y30A/K137A mutant protein, demonstrating the important roles of the two amino acids in cap-recognition.

In conclusion, we have characterized the structural features of SARS-CoV-2 nsp10/nsp16 2′-O-methylase at the atomic level. The conserved SAM-binding pocket could be targeted by SAM-analogs such as SIN. In addition, the plastic feature observed for the RNA-binding groove might provide new opportunities for antiviral drug design. Finally, the similar overall structures, conserved SAM-binding pockets, and resembled nsp10/nsp16 binding interface shared by the nsp10/nsp16 2′-O-methylases of SARS-CoV-2, SARS-CoV, and MERS-CoV highlight that these enzymes are unlikely associated with the different pathogenic behaviors of these coronaviruses. The difference in the pathogenesis and transmission-capacity between SARS-CoV-2 and other coronaviruses should be related to the unique structural features and functional elements of other viral proteins. E.g., SARS-CoV-2 spike has been shown to possess an apparently higher affinity for human receptor ACE2 than that observed for SARS-CoV spike.^5^

## Supplementary information

Supplementary Materials

## Data Availability

The data sets used and/or analyzed during the current study are available from the corresponding author on reasonable request. Atomic coordinates and structure factors of the reported crystal structures have been deposited into the Protein Data Bank (https://www.rcsb.org; PDB: 7C2I, 7C2J).
