# Supplementary material for: Crystal structure of SARS-CoV-2 nsp10/nsp16 2′-O-methylase and its implication on antiviral drug design
Source: Signal Transduct Target Ther. 2020 Jul 29;5:131. doi: 10.1038/s41392-020-00241-4 (PMC7388121; doi:10.1038/s41392-020-00241-4)
Supplement: Supplementary file 1 — Supplementary Materials [file 41392_2020_241_MOESM1_ESM.docx]

Supplementary Materials for

**Crystal structure of SARS-CoV-2 nsp10/nsp16 2’-O-methylase and its implication on antiviral drug design**

Sheng Lin^1#^, Hua Chen^1#^, Fei Ye^1^, Zimin Chen^1^, Fanli Yang^1^, Yue Zheng^1^, Yu Cao^1,2^, Jingxin Qiao^1^, Shengyong Yang^1^, Guangwen Lu^1*^

^1^ West China Hospital Emergency Department (WCHED), State Key Laboratory of Biotherapy and Cancer Center, West China Hospital and Collaborative Innovation Center of Biotherapy, Sichuan University, 610041 Chengdu, Sichuan, China

^2^ Disaster Medicine Center, West China Hospital, Sichuan University, 610041 Chengdu, Sichuan, China

^#^These authors contributed equally to this work.

*Correspondence to: Guangwen Lu ([lugw@scu.edu.cn](mailto:lugw@scu.edu.cn))

**This PDF file includes:**

Materials and Methods

Fig. S1 to S4

Table S1

**Materials and Methods**

**Cloning, Expression and Purification**

The coding sequence of nsp10 and nsp16 from the novel human coronavirus (designated as HCoV-19^1^ or SARS-CoV-2^2^, GenBank: MN908947.3) were synthesized (Convenience Biology Corporation) and sub-cloned into pGEX-6p-1 and pET-30a, respectively. The nsp16 mutant (Y30A/K137A) was generated with a standard two-step PCR-based strategy. For protein expression, the plasmids of nsp10 (with an N-terminal GST tag) and nsp16 (wild type and mutant, with an N-terminal 6×His tag) were co-transformed into *E.coli* BL21 (DE3). The cells containing both plasmids were then grown in LB medium supplemented with 50 µg/ml ampicillin and 25 µg/ml kanamycin at 37 °C and induced for protein expression with 400 µM isopropyl-β-D-thiogalactopyranoside (IPTG) at 16 °C for about 12 hours. Cells were harvested, lysed by sonication in re-suspension buffer consisting of 20 mM Hepes (pH 7.5) and 500 mM NaCl, and clarified via centrifugation at 18000×g for 30 minutes. Cleared lysate-supernatant was bound to Glutathione-Sepharose resin (GE Healthcare) to remove the contaminated proteins, and cleaved by on-column digestion using PreScission Protease to remove the GST tag. The target proteins were then collected and further purified by gel filtration in re-suspension buffer using a Superdex 200 Increase 10/300 GL column (GE Healthcare).

To remove pre-bound SAM that was simultaneously captured by nsp10/nsp16 during protein co-expression, the complex protein derived directly from *E. coli* was first denatured using a buffer consisting of 6 M Guanidine-HCl, 50 mM Tris-HCl (pH 8.0), 100 mM NaCl, 10 mM DTT and 10 % Glycerol at a final concentration of 10 mg/ml. The denatured protein was then refolded at 4 °C by dialyzing against a buffer containing 100 mM Tris-HCl (pH 8.0), 400 mM L-Arg-HCl, 5 mM reduced glutathione, 0.5 mM oxidized glutathione and 0.5 mM ZnCl_2_ for about 10 hours. Subsequently, the refolded protein was concentrated using an Amicon Stirred Cell concentrator (Merck Millipore) with a 10-kDa cutoff membrane and then adjusted to re-suspension buffer. The protein heterodimer was then further puriﬁed by gel ﬁltration using a Superdex 200 Increase 10/300 GL column (GE Healthcare).

**Crystallization**

Commercial crystallization kits (Molecular Dimensions and Hampton Research) were used for crystallization screening by the vapour-diffusion sitting-drop method. In brief, 1 µl co-expressed nsp10/nsp16 complex (with additional SAM (Sigma) at a 1:5 protein-to-SAM molar ratio or without additional SAM) was mixed with 1 µl reservoir solution. The resultant mixture was then equilibrated against 70 µl reservoir solution at 18 °C. Diffractable crystals of the nsp10/nsp16 complex without additional SAM were obtained in a condition consisting of 0.1 M MES (pH 6.5) and 12 % w/v PEG 20000, with a protein concentration of 4.8 mg/ml. Crystals of the complex with additional SAM were obtained in a condition consisting of 0.4 M Sodium malonate (pH 6.0), 0.1 M MES (pH 6.0), and 0.5% w/v PEG 10000, with a final protein concentration of 2.4 mg/ml.

**Data collection and structure determination**

For data collection, crystals were ﬂash-cooled in liquid nitrogen after a brief soaking in reservoir solution supplemented with 20% (v/v) glycerol. Diffraction data were collected at Shanghai Synchrotron Radiation Facility (SSRF) beamline BL19U1^3^. The collected data were then processed with HKL2000^4^ for indexing, integration and scaling. The two structures reported in this study were both solved by molecular replacement with the Phaser^5^ program from the CCP4 suite^6^, using the structure of SARS-CoV nsp10/nsp16 (PDB: 3R24)^7^ as the search model. Initial restrained rigid-body refinement was performed using Refmac5^8^, which was followed by manual rebuilding and adjustment in Coot^9^. The SAM molecule was manually built using Coot based on the simulated annealing omit Fo-Fc maps. And water molecules were automatically added in Phenix.refine^10^. The stereochemical quality of the final models were assessed through the program Procheck^11^. Final statistics for data collection and structure refinement are summarized in Table S1. All structural figures were generated using Pymol (http:// [www.pymol.org](http://www.pymol.org)).

**DSF (Differential Scanning Fluorimetry) assay**

The differential scanning fluorimetry assay was performed as reported^12^. 5×SYPRO Orange dye (from a stock concentration of 5000×, Sigma) was used to probe protein thermal denaturation. Optimal condition was achieved using a buffer containing 100 mM Tris-HCl (pH 7.5), and 500 mM NaCl. The nsp10/nsp16 complex (co-expression or refolded) was pre-mixed with SAM (Sigma) or SIN (an SAM analog that has been shown to inhibit multiple viral MTases^13-16^, J&K Scientific) to yield a final protein concentration of 10 µM and ligand concentration of 200 µM. For the DSF test, 15 µl of the sample (nsp10/nsp16 alone, nsp10/nsp16 with SAM, and nsp10/nsp16 with SIN) was heated using a linear temperature-gradient of 25-95 °C in 75 minutes using CFX Connect Real-Time System (Bio-Rad). The fluorescence signal as a function of temperature was monitored continuously. Each sample was measured in triplet and fitted with the Boltzmann equation using GraphPad Prism 5 (GraphPad Software).

**ITC (Isothermal Titration Calorimetry) assay**

The ITC measurements were performed at 25 °C on a Microcal PEAQ-ITC titration calorimeter (Malvern) with a reference power of 5 µcal/s and stirring speed of 750 rpm.

Proteins (co-expression and refolded) and ligands (SAM, SIN and ^7Me^GpppA (NEB)) were first prepared in the ITC buffer consisting of 100 mM Tris-HCl (pH 7.5) and 500 mM NaCl. The protein- and ligand-concentrations used for titration are: 400 µM SAM to 50 µM co-expressed nsp10/nsp16 protein, 650 µM SIN to 40 µM co-expressed nsp10/nsp16 protein, 700 µM SAM to 40 µM refolded nsp10/nsp16 protein, and 470 µM SIN to 20 µM refolded nsp10/nsp16 protein, 600 µM ^7Me^GpppA to 35 µM co-expressed nsp10/nsp16 protein, 600 µM ^7Me^GpppA to 35 µM co-expressed nsp10/nsp16 (Y30A/K137A) protein. All samples were centrifuged at 18000×g for 20 min before titration. Each titration typically involves 19 injections of 2 µl ligand with 4 s durations and 120 s intervals. The data fitting and analyses were performed using the PEAQ-ITC analysis software package provided by MicroCal.

**References:**

1. Jiang, S. et al. A distinct name is needed for the new coronavirus. *Lancet* **395**, 949 (2020).

2. Coronaviridae Study Group of the International Committee on Taxonomy of, V. The species Severe acute respiratory syndrome-related coronavirus: classifying 2019-nCoV and naming it SARS-CoV-2. *Nat Microbiol* **5**, 536-544 (2020).

3. Zhang, W.-Z. et al. The protein complex crystallography beamline (BL19U1) at the Shanghai Synchrotron Radiation Facility. *Nuclear Science and Techniques* **30**, 170 (2019).

4. Otwinowski, Z. & Minor, W. Processing of X-ray diffraction data collected in oscillation mode. *Methods Enzymol* **276**, 307-326 (1997).

5. Read, R.J. Pushing the boundaries of molecular replacement with maximum likelihood. *Acta Crystallogr D Biol Crystallogr* **57**, 1373-1382 (2001).

6. Collaborative, C.P.J.A.c.S.D., Biological crystallography The CCP4 suite: programs for protein crystallography. *Acta Crystallogr D Biol Crystallogr* **50**, 760 (1994).

7. Chen, Y. et al. Biochemical and structural insights into the mechanisms of SARS coronavirus RNA ribose 2'-O-methylation by nsp16/nsp10 protein complex. *Plos Pathog* **7**, e1002294 (2011).

8. Murshudov, G.N. et al. REFMAC5 for the refinement of macromolecular crystal structures. *Acta Crystallogr D Biol Crystallogr* **67**, 355-367 (2011).

9. Debreczeni, J.E. & Emsley, P. Handling ligands with Coot. *Acta Crystallogr D Biol Crystallogr* **68**, 425-430 (2012).

10. Adams, P.D. et al. PHENIX: a comprehensive Python-based system for macromolecular structure solution. *Acta Crystallogr D Biol Crystallogr* **66**, 213-221 (2010).

11. Laskowski, R.A., Macarthur, M.W., Moss, D.S. & Thornton, J.M. PROCHECK: a program to check the stereochemical quality of protein structures. *J Appl Crystallogr* **26**, 283-291 (1993).

12. Cao, X. et al. Molecular mechanism of divalent-metal-induced activation of NS3 helicase and insights into Zika virus inhibitor design. *Nucleic Acids Res* **44**, 10505-10514 (2016).

13. Pugh, C.S., Borchardt, R.T. & Stone, H.O. Sinefungin, a potent inhibitor of virion mRNA(guanine-7-)-methyltransferase, mRNA(nucleoside-2'-)-methyltransferase, and viral multiplication. *J Biol Chem* **253**, 4075-4077 (1978).

14. Dong, H. et al. West Nile virus methyltransferase catalyzes two methylations of the viral RNA cap through a substrate-repositioning mechanism. *J Virol* **82**, 4295-4307 (2008).

15. Barral, K. et al. Development of specific dengue virus 2'-O- and N7-methyltransferase assays for antiviral drug screening. *Antiviral Res* **99**, 292-300 (2013).

16. Aouadi, W. et al. Binding of the Methyl Donor S-Adenosyl-l-Methionine to Middle East Respiratory Syndrome Coronavirus 2'-O-Methyltransferase nsp16 Promotes Recruitment of the Allosteric Activator nsp10. *J Virol* **91**, e02217-16 (2017).

Fig. S1


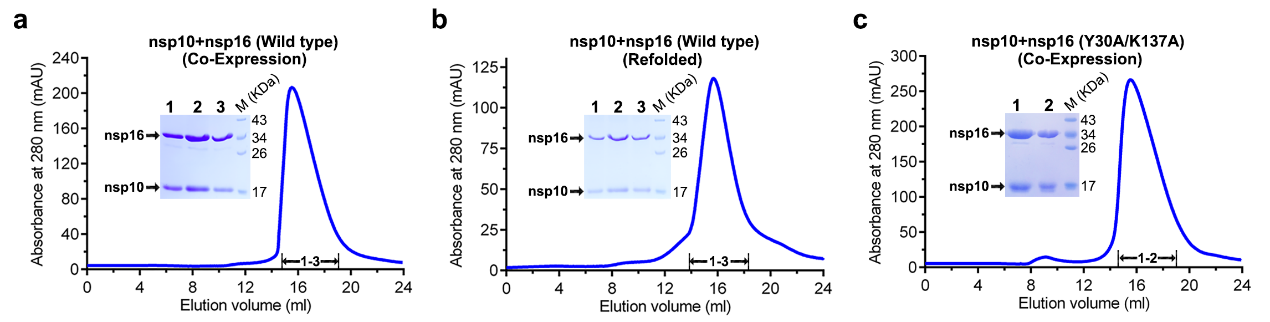


**Fig. S1. Solution behavior of SARS-CoV-2 nsp10/nsp16 complex on a Superdex 200 Increase 10/300 GL column.** The inset figure shows the SDS-PAGE analyses of the pooled samples. **a** The protein-complex purified directly from *E. coli* (designated as co-expression). **b** The protein-complex after the denaturing-refolding cycle (designated as refolded). **c** The protein-complex of wild-type nsp10 with nsp16-Y30A/K137A mutant (designated as Y30A/K137A co-expression) purified directly from *E. coli*.

Fig. S2


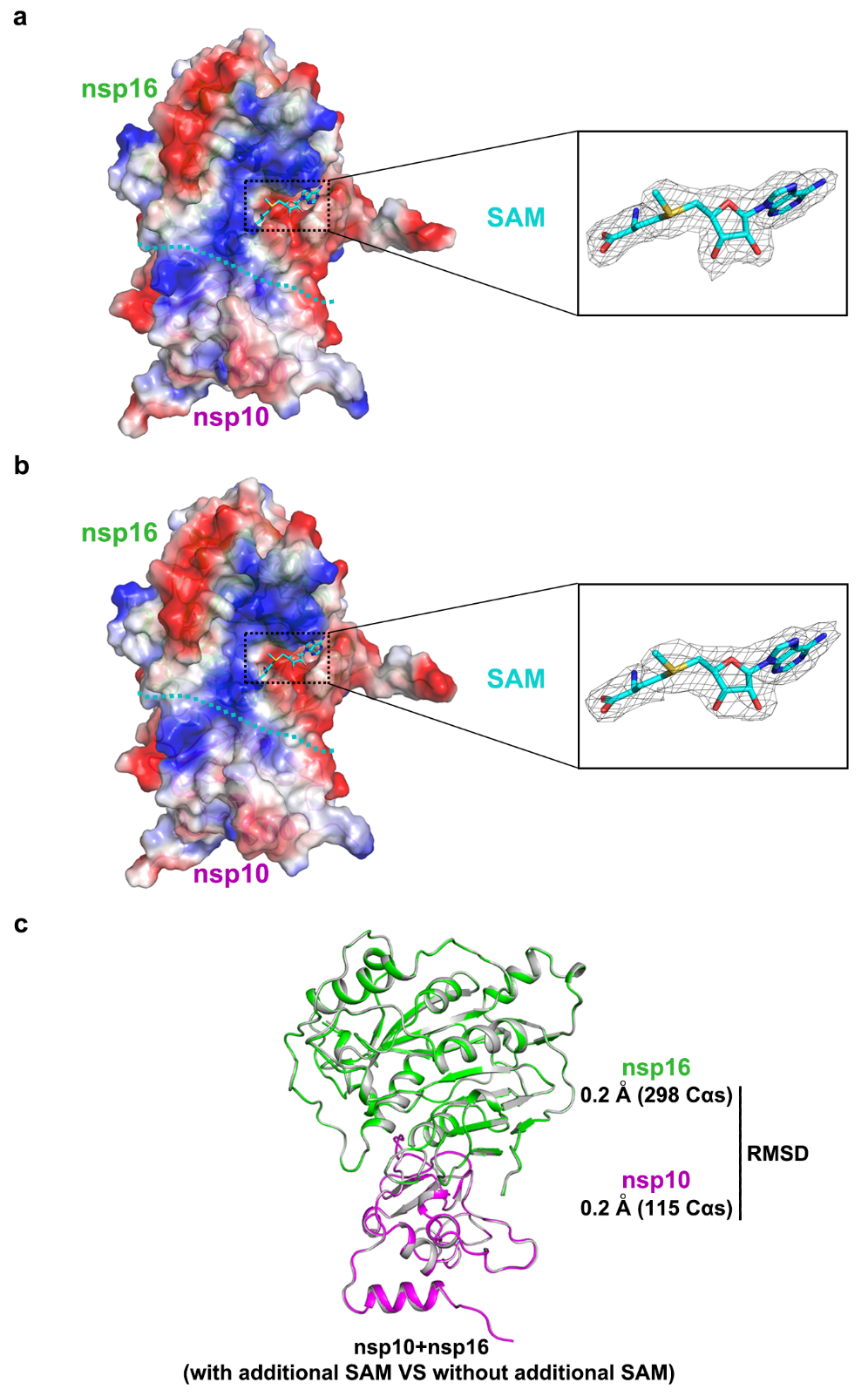


**Fig. S2. Structure of SARS-CoV-2 nsp10/nsp16 hetero-dimer solved from crystals obtained with additional SAM and without additional SAM.** The structures are presented by depicting a full electrostatic surface of the complex. The rough boundary between nsp10 and nsp16 is marked with a dashed line. The bound SAM molecule, whose electron densities are contoured at 1.0 σ using the 2｜Fo｜-｜Fc｜map, is shown in the right panel. **a** Structure solved from crystals obtained with additional SAM. **b** Structure solved from crystals obtained without additional SAM. **c** Superimposition of the structure with additional SAM (green for nsp16 and magenta for nsp10) onto that without additional SAM (gray). The calculated RMSD values are shown.

Fig. S3


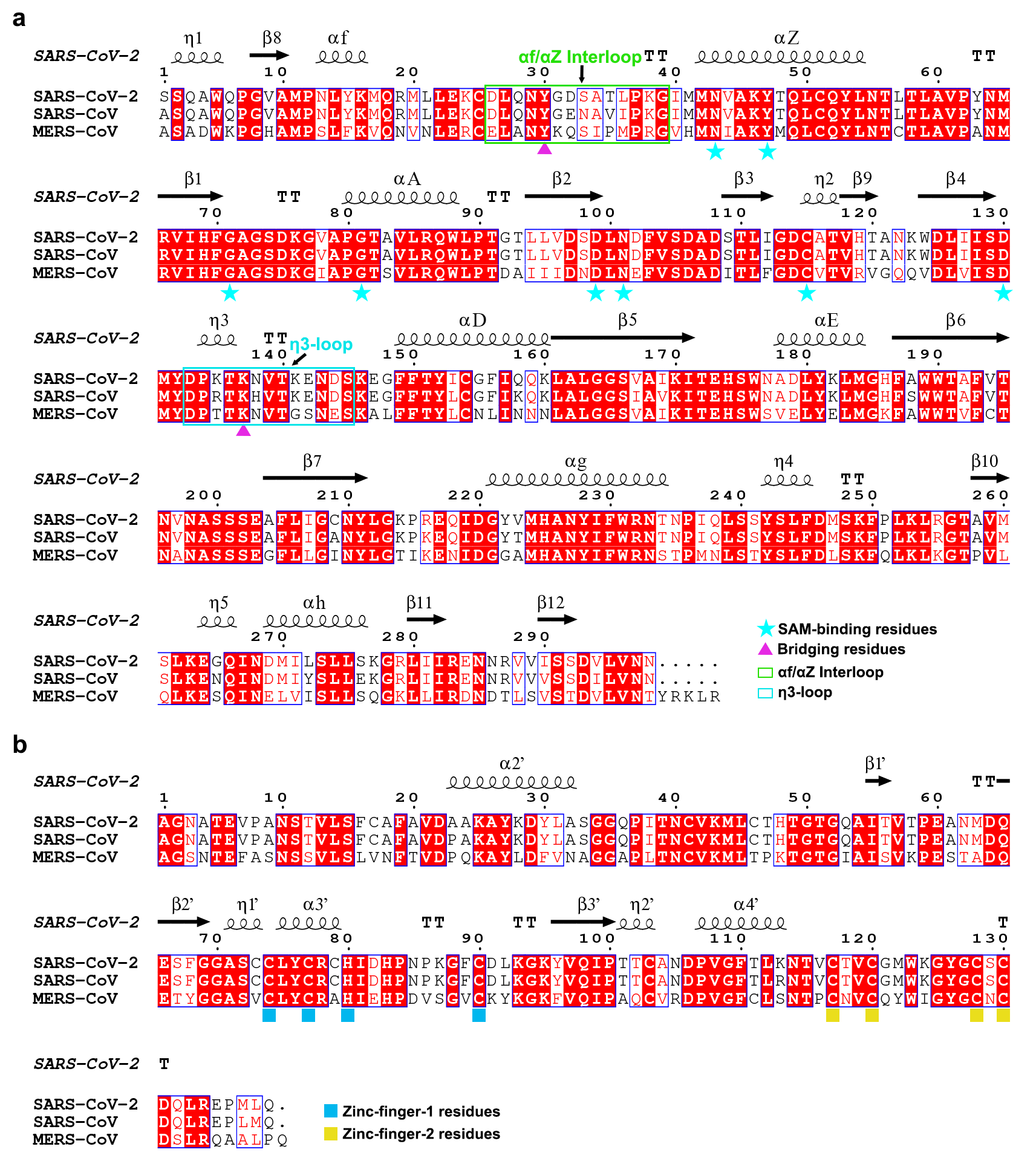


**Fig. S3. Structure-based multiple sequence alignment of the nsp10 and nsp16 proteins from SARS-CoV-2, SARS-CoV, and MERS-CoV.** The horizontal arrows indicate β-strands, and the spinal lines represent α-helices or 3_10_ helices. **a** Multiple sequence alignment for nsp16. Those residues interacting with SAM are marked with stars. The αf/αZ interloop and the η3-loop that line the RNA-binding groove are highlighted by encircling with green and cyan rectangle, respectively. Residues Y30 and K137 that are observed to form an inter-groove bridge in our structure are marked with triangles. **b** Multiple sequence alignment for nsp10. Those residues involved in zinc-coordination are marked with squares.

Fig. S4


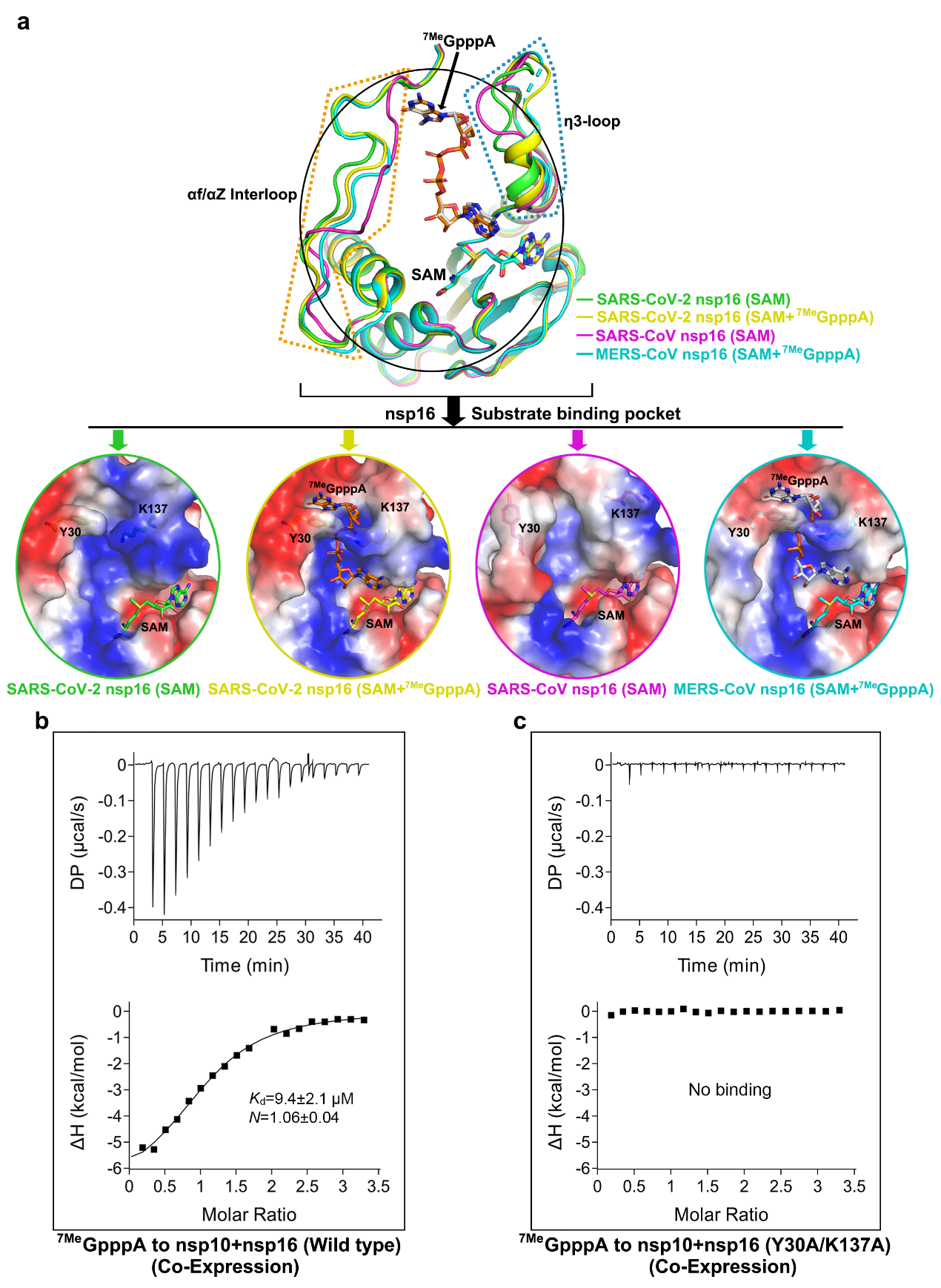


**Fig. S4. A magnified view of the cap-RNA binding groove. a** Superimposition of the structures of SARS-CoV-2 nsp10/nsp16 (green color), SARS-CoV nsp10/nsp16 (magenta color, PDB: 3R24), MERS-CoV nsp10/nsp16 with a bound RNA-cap substrate (cyan color, PDB: 5YNM), and SARS-CoV-2 nsp10/nsp16 in complex with ^7Me^GpppA RNA-cap (yellow color, PDB: 6WKS). A magnified view on the RNA-binding groove is shown in the upper panel. The groove in the individual structure is further highlighted in the bottom panel by showing the electrostatic surface. Residues Y30 and K137, which are observed to form an inter-groove bridge in our structure and in the MERS-CoV and SARS-CoV-2 ternary complex structures but not in the SARS-CoV structure, are labeled. Clearly shown is that our structure more parallels to the structures of MERS-CoV and SARS-CoV-2 nsp10/nsp16 with the bound RNA-cap substrate than to the SARS-CoV nsp10/nsp16 structure whose RNA-binding groove is completely open. **b** Binding of ^7Me^GpppA to wild-type SARS-CoV-2 nsp10/nsp16 characterized by ITC. **c** Binding of ^7Me^GpppA to SARS-CoV-2 nsp10/nsp16 Y30A/K137A mutant characterized by ITC.

Table S1

**Table S1** Data collection and structure refinement statistics

|  | nsp10+nsp16  (with additional SAM) | nsp10+nsp16  (without additional SAM) |
| --- | --- | --- |
| **Data collection** |  |  |
| Space group | P3121 | P3121 |
| Cell dimensions |  |  |
| *a*, *b*, *c* (Å) | 167.82, 167.82, 51.57 | 167.53, 167.53, 51.69 |
| α, β, γ (°)  Wavelength (Å) | 90.00, 90.00, 120.00  0.97852 | 90.00, 90.00, 120.00  0.97852 |
| Resolution (Å) | 50.00-2.50 (2.59-2.50) | 50.00-2.80 (2.90-2.80) |
| *R*_merge_ | 0.204 (0.937) | 0.295 (0.918) |
| *I* / σ*I* | 10.67 (1.67) | 6.67 (2.00) |
| Completeness (%) | 100.0 (100.0) | 99.9 (100.0) |
| Redundancy | 10.9 (9.8) | 6.8 (6.9) |
|  |  |  |
| **Refinement** |  |  |
| Resolution (Å) | 43.94-2.50 | 41.88-2.80 |
| No. reflections | 29023 | 20712 |
| *R*_work_ / *R*_free_ | 0.1744/0.2089 | 0.2025/0.2355 |
| No. atoms |  |  |
| Protein | 3196 | 3191 |
| Ligand/ion | 29 | 29 |
| Water | 164 | 103 |
| *B*-factors |  |  |
| Protein | 31.7 | 24.4 |
| Ligand/ion | 24.9 | 17.3 |
| Water | 32.6 | 18.4 |
| R.m.s. deviations |  |  |
| Bond lengths (Å) | 0.009 | 0.007 |
| Bond angles (°) | 0.891 | 0.713 |
| Ramachandran plot (%)  Favored region  Allowed region  Outlier region  PDB code | 97.56  2.44  0.00  7C2I | 96.33  3.67  0.00  7C2J |

In each case, a single crystal was used to collect the data. Values in parentheses are for the highest-resolution shell.
